# Supplementary material for: Effect of the COVID-19 pandemic on health facility delivery in Ethiopia; results from PMA Ethiopia’s longitudinal panel
Source: PLOS Glob Public Health. 2021 Oct 13;1(10):e0000023. doi: 10.1371/journal.pgph.0000023 (PMC10021675; doi:10.1371/journal.pgph.0000023)
Supplement: S1 Table — (PDF) [file pgph.0000023.s001.pdf]

Appendix Table A: Reasons given for how COVID-19 affected delivery location		
	Weighted N=46	
	n	%
Fear of COVID-19 transmission	33	71.3
Fear would be alone during delivery	19	42.4
No transportation	16	35.9
Concerned no beds available	7	15.9
Health facility closed	6	13.1
Restrictions on movement	4	9.1
Unable to afford services	1	2.5
Partner disapproval	0	0

*Women could select multiple options and thus totals do not add to 100%
