# Supplementary material for: Effect of the COVID-19 pandemic on health facility delivery in Ethiopia; results from PMA Ethiopia’s longitudinal panel
Source: PLOS Glob Public Health. 2021 Oct 13;1(10):e0000023. doi: 10.1371/journal.pgph.0000023 (PMC10021675; doi:10.1371/journal.pgph.0000023)
Supplement: Attachment — Submitted filename: Response to Reviewers.docx [file pgph.0000023.s002.docx]

Thank you for the opportunity to revise and resubmit our manuscript. We have made edits per the recommendations of reviewers and thank them for their thorough review. Please find our point-by-point response below.

Reviewer #1: Dear Authors,
thank you very much for the interesting and important work.

Response: Thank you to the reviewer.

Very few comments:
In the introduction you could as well relate to the MMR in Sierra Leone during the Ebola Crisis - to emphasise the importance of "side Effects" of an empidemic.

Response: Thank you for this suggestion. We have added citations referencing the detrimental effect of Ebola to maternal and newborn health on page 6.

“This is especially challenging in low- and middle-income countries where population-level data on the effects of COVID-19 on MNH care-seeking and health behaviors are extremely limited, but where evidence from previous pandemics has found serious consequences for maternal and newborn health, including increased maternal and newborn morbidity and mortality and reductions in health seeking behaviors^6–8^”

On page 14 in the 4th last line you used the verb developing instead of delivering.

Response: Thank you. This has been revised.

Reviewer #2: Review Comments:
Ø Abstract, settings sub-section
Covering 91% of the population. However, on page 10, dependent variable sub-section, the footnote shows that the PMA Ethiopia conducted in 6 regions with a total coverage of 90% of the population of the country. So, which figure is indicated the coverage rate of your population frame?

Response: Thank you. We have updated the footnote to reflect approximately 91% of the population is covered by the sampling frame.

Ø Independent variables sub-section, page 10/11
The variable of primary interest, which has a binary nature, of woman delivered prior to or during COVID-19 pandemic. Is it your variable of interest (dependent variable) or independent?
Since, the independent variables are listed as covariates (age, education, wealth …). To mean that independent variables are covariates.

Response: We have relabeled the covariates section to indicate our primary independent variable of interest is delivery relative to the COVID-19 pandemic and the remaining variables are adjustment covariates.

Ø Table 1, p-value=0.00, it better to use, p-value<=0.01

Response: This has been updated.

Ø Table 1, rural row vs urban column, the figure shows 100, recheck it again, maybe it is 0.0.

Response: Thank you. This has been updated.
